# The Role of Bladder-Washing Cytology as an Adjunctive Method to Cystoscopy During Follow-Up for Low-Grade TaT1 Non-Muscle-Invasive Bladder Cancer

**DOI:** 10.3390/cancers16213708

**Published:** 2024-11-01

**Authors:** Enric Carbonell, Clàudia Mercader, Héctor Alfambra, Paulette Narvaez, Eric Villalba, Rita Pagès, Ignacio Asiain, Meritxell Costa, Agustín Franco, Antonio Alcaraz, María José Ribal, Antoni Vilaseca

**Affiliations:** Department of Urology, Hospital Clínic de Barcelona, Villarroel 170, 08036 Barcelona, Spain; encarbonell@clinic.cat (E.C.);

**Keywords:** non-muscle-invasive bladder cancer, urine cytology, urothelial carcinoma, cystoscopy, follow-up

## Abstract

Background and Objective: The role of urine cytology during follow-up for low-grade (LG) non-muscle-invasive bladder cancer (NMIBC) is not well established, although cytology has low sensitivity in detecting LG recurrences. Our study aims to evaluate the impact of urine cytology as a complementary method to cystoscopy during follow-up for LG NMIBC. Methods: Patients diagnosed with primary LG TaT1 bladder cancer (BC) between 2010 and 2020 were included. Patients were stratified according to the EAU NMIBC scoring model. Urine cytology was performed during follow-up cystoscopy. The outcomes of the study were BC recurrence and upgrading to high-grade (HG). Cytology utility was established by assessing whether its result led to management change. Results: We included 337 patients with LG TaT1 BC. EAU risk group distribution was low in 262 (77.7%), intermediate in 57 (16.9%), and high-risk in 18 (5.3%) cases. With a median follow-up of 5 years, 166 (49.3%) patients experienced recurrence. Cystoscopy was positive in 154 (92.8%) and suspicious in 12 (7.2%) cases. Urine cytology was positive in 33 (19.9%) cases but only changed management in 3 (0.89%), all with suspicious cystoscopy. Positive cytology at first recurrence was associated with higher risk of upgrading during follow-up (HR 2.781, *p* = 0.006) and lower upgrading-free survival (*p* = 0.001). Conclusions: The role of urine cytology to detect first recurrences during follow-up for primary LG TaT1 NMIBC might be limited to patients with non-conclusive lesions in the cystoscopy. A positive cytology at first recurrence is associated with a higher risk of upgrading to HG BC during follow-up.

## Simple Summary

Follow-up protocols for non-muscle-invasive bladder cancer (NMIBC) include mainly cystoscopy and urinary cytology. However, the role of urine cytology during follow-up for low-grade (LG) NMIBC is not entirely known, and studies assessing adherence to guideline recommendations have revealed an overuse of urinary cytology. Our aim with this study was to evaluate the impact of urine cytology as a complementary method to cystoscopy during follow-up for LG NMIBC. We found that performing urine cytology during follow-up for primary LG non-muscle-invasive bladder cancer is only useful to detect recurrences when suspicious lesions are seen during cystoscopic evaluation. Nevertheless, a positive cytology alerts to the risk of progression to high-grade disease during surveillance.

## 1. Introduction

Follow-up protocols for non-muscle-invasive bladder cancer (NMIBC) include mainly cystoscopy and urinary cytology along with upper-tract imaging performed periodically. Surveillance protocols for NMIBC proposed by both the European Association of Urology (EAU) and the American Urological Association (AUA) guidelines recommend different follow-up schemes based on NMIBC risk categories [1,2]. Despite being an invasive test, cystoscopy remains the cornerstone of NMIBC diagnosis and follow-up, as to date no urinary biomarker has been accepted nor recommended in routine practice to replace cystoscopy [3]. The addition of urine cytology apart from cystoscopy also has a role in NMIBC follow-up, as it has good sensitivity detecting high-grade (HG) tumours and carcinoma in situ (CIS), which can be missed by cystoscopy, and alerting to the presence of urothelial carcinoma (UC) in the upper urinary tract [4]. Nevertheless, cytology has limitations in detecting low-grade (LG) disease, with sensitivity as low as 16% [4]. The EAU only recommend cytology as an adjunct to cystoscopy in high-risk NMIBC, and the AUA guidelines recommend it only for both high- and intermediate-risk patients for early detection of HG recurrences [1]. However, with the 2021 EAU NMIBC scoring model, patients with LG tumours and additional risk factors can be classified as high-risk individuals. In this setting, information regarding the usefulness of cytology is scarce. Regarding low-risk patients with mostly LG tumours that rarely progress to HG disease or, when presenting recurrences, are most likely to have LG non-invasive tumours, urinary cytology is not included in the follow-up scheme [5,6]. Surprisingly, studies assessing adherence to guideline recommendations reveal an overuse of urinary cytology during follow-up for low-risk or LG disease [7,8]. Bree et al. report that the use of urinary cytology or other urinary biomarkers increased from 44.8% in 2004 to 54.9% in 2013 in the LG follow-up scenario [7]. Similarly, a recent survey of 747 members of the Society of Urologic Oncology (SUO) reveals that 53% of the respondents routinely perform urinary cytology during surveillance of low-risk NMIBC [8].

The aim of the present study is to assess the role of bladder-washing cytology as a complementary method to cystoscopy during surveillance of LG TaT1 NMIBC.

## 2. Materials and Methods

After approval from our institutional review board (IRB) (HCB/2023/0388), we reviewed data prospectively collected from patients diagnosed with debut LG Ta or T1 bladder cancer (BC) after transurethral resection of bladder tumour (TURBT) between 2010 and 2020 at our institution. We excluded patients with concomitant CIS in bladder biopsies, with upstaging or upgrading after re-TURBT, with pTx staging, with previous history of upper-tract urothelial carcinoma (UTUC), patients missing urine cytology during follow-up, and those with less than 1 year of follow-up. Data were prospectively collected in an ethics-approved, secure database, and included demographics, baseline clinicopathologic parameters, initial diagnosis pathology after TURBT, cystoscopy, and cytology information during surveillance and recurrence patterns during follow-up. All patients, for the purpose of this study, were re-stratified into risk groups according to the 2021 EAU NMIBC scoring model [1]. Patients diagnosed before 2021 were previously followed-up with according to the scoring model being used at that moment and our institutional protocols. During the study period, we routinely performed urine cytology for NMIBC surveillance in all risk groups due to research being done in our department with urinary markers. We use bladder-washing urine cytology in our clinical practice due to our experience getting the best specimens for analysis according to our uro-pathologists. However, there is no evidence for or recommendation in the EAU guidelines supporting this fact in comparison with voided urine. All patients underwent a first cystoscopy and cytology after 3 months of the initial TURBT. Then, low-risk patients underwent cystoscopy and cytology every 6 months for 2 years and annually thereafter till completing 5 years. In intermediate-risk patients, cystoscopy and cytology were performed every 6 months for 5 years and annually thereafter till completing 10 years. High-risk patients underwent a stricter protocol with cystoscopy and cytology every 3 months for 2 years, then every 6 months till completing 5 years and annually thereafter. Patients received intravesical therapy with either mitomycin C or bacillus Calmette–Guérin (BCG) according to our institution protocols. Surveillance cystoscopy findings were grouped into three categories (negative, suspicious, and positive) according to the treating physician’s notes. Urine cytology results were reported with standardized nomenclature from 2016 onwards according to the Paris working group [9]. For the purpose of the study, we reclassified cytology results into three groups: negative, if negative or only atypia were described; positive, if suspicion for urothelial carcinoma or malignancy was reported; and non-diagnostic, if no adequate diagnosis was possible.

The main outcomes were BC recurrence after initial diagnosis and progression to HG disease. Recurrence was defined as a positive pathology report from a TURBT specimen or an outpatient, office-based biopsy. Patients diagnosed with papillary urothelial neoplasm of low malignant potential were considered LG recurrences. The clinical impact of cytology as an adjunctive to cystoscopy during the surveillance of LG tumours was assessed by analysing the change in the follow-up/management protocol triggered by a positive cytology that resulted in recurrence detection.

Demographic and clinicopathologic variables were assessed with frequencies and proportions for categorical variables, while medians and interquartile ranges (IQR) were used for continuous variables. Variables stratified by EAU risk groups were compared using the Chi-square test for categorical variables and Kruskal–Wallis for continuous variables. Fisher exact tests were used when cells had <5 cases. Cox regression models were performed to assess the association between a positive cytology at first recurrence and progression to HG BC during follow-up. Adjustment was made for the EAU prognostic factor risk groups which consider age, multifocality, tumour size, stage, and grade. Upgrade-free survival to HG urothelial carcinoma was represented using the Kaplan–Meier method. Survival curves were represented stratified by cytology result and compared using the two-sided log-rank test. A double-sided *p* value < 0.05 was considered statistically significant. The data were analysed using IBM SPSS Statistics (Version 25.0) software.

## 3. Results

### 3.1. Descriptive Characteristics

**Figure 1:**
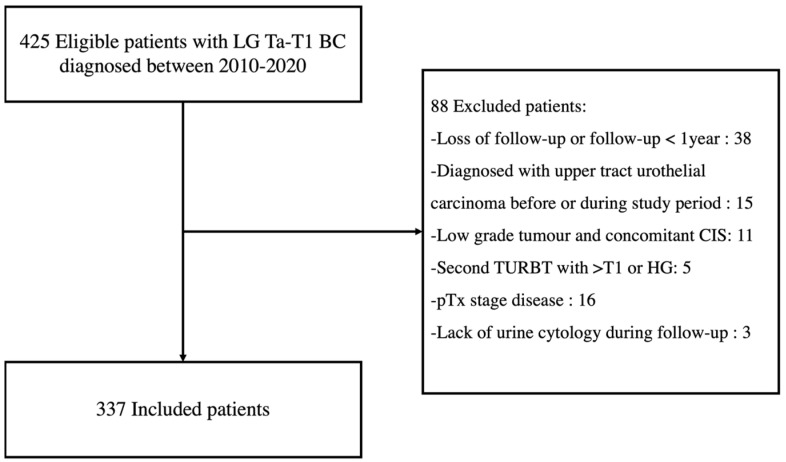
Flowchart of patient inclusion. LG = Low-grade; BC = Bladder Cancer; CIS = Carcinoma in situ; TURBT = Transurethral resection of bladder tumour; HG = High-grade.

We identified 425 eligible patients diagnosed with LG Ta-T1 BC between 2010 and 2020, and 337 were included for analysis (Figure 1).

Baseline clinicopathological characteristics of included patients diagnosed with LG Ta-T1 BC are summarised in Table 1. Median age at diagnosis was 68 years old (interquartile range (IQR) 59–78 years) and most patients were male 268 (79.5%). Initial pT stage after TURBT was Ta in 299 (88.7%) and T1 in 38 (11.3%). Initial EAU NMIBC risk group distribution was low in 262 (77.7%), intermediate in 57 (16.9%) and high-risk in 18 (5.3%) patients. Twenty of the included patients received adjuvant intravesical mitomycin C and 4 BCG after LG NMIBC diagnosis.

### 3.2. Recurrence Patterns and Predictive Value of Urine Cytology

With a median follow-up of 5 years (IQR 3.6–6.7 years) after initial LG NMIBC diagnosis, 166 (49.3%) patients experienced BC recurrence (Table 2). Median time to recurrence after initial diagnosis was 12 months (IQR 6–23 months). Cystoscopy at first recurrence was positive in 154 (92.8%) of the recurrent cases and suspicious in 12 (7.2%) (Figure S1); no patients were diagnosed with recurrence with a negative cystoscopy. Urine cytology was positive in 33 (19.9%) patients at first BC recurrence. Urine cytology at recurrence was positive in 18.3% patients with initial low-risk disease, in 19.4% with intermediate-risk BC, and in 44.4% with high-risk BC (*p* = 0.443). Pathology characteristics at first recurrence are shown in Table S1. Most of the first recurrences after initial diagnosis were non-invasive Ta (80.7%) or T1 (9.0%) lesions of LG (89.2%). Five patients were diagnosed with papillary urothelial neoplasm of low malignant potential that were considered as LG recurrences. In 18 (10.8%) patients with initial LG NMIBC, there was an upgrading to HG disease at first recurrence. In 10 (55.6%) cases of upgrading at first recurrence, cytology was positive. During follow-up, 36 patients (21.7%) progressed to HG disease and 21 (12.7%) patients experienced upstaging. As expected, upgrading or upstaging were more frequent in the intermediate- and high-risk groups (*p* = 0.018 and *p* = 0.002, respectively).

**Figure 2:**
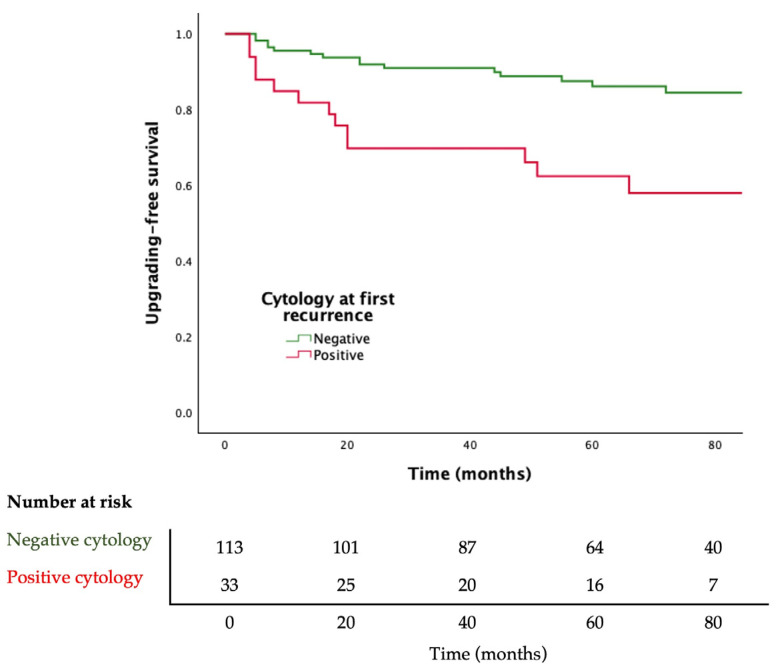
Kaplan–Meier curves for progression to high-grade bladder cancer stratified by urine cytology result. Log-rank *p* = 0.001.

Adjusted Cox regression analysis showed that a positive urine cytology at first recurrence of BC is associated with upgrading to HG disease during follow-up (HR 2.781; 95% CI 1.335–5.794, *p* = 0.006). Moreover, patients with positive cytology at first recurrence of BC have lower upgrading-free survival (log rank *p* = 0.001) (Figure 2).

### 3.3. Role of Bladder-Washing Cytology as an Adjunctive Method to Cystoscopy During Follow-Up of LG NMIBC

Bladder-washing urinary cytology changed management and led to recurrence diagnosis in 3 (0.89%) patients during the study period. In all of them, a suspicious area or lesion was found during cystoscopic examination, and a positive urinary cytology influenced subsequent management (Table 3). In two cases, a suspicious image on cystoscopy lead to the scheduling of an early cystoscopy to decide if the suspected area had changed appearance. The positive cytologic result led to a resection or biopsy of the suspected area that was positive for BC. In the remaining case, a suspicious papillary lesion was found, and an outpatient appointment was set to decide how to proceed with the cytology result. Cytology was positive and a TURBT of the lesion was performed with a pathology report showing HG BC.

## 4. Discussion

Surveillance of patients diagnosed with NMIBC should be scheduled according to the risk of recurrence or progression after initial treatment. Several prognostic models have been proposed, with the EAU NMIBC prognostic factor risk groups and the AUA risk stratification being the most widely used [1,2]. However, despite patient stratification into risk groups, follow-up protocols for each risk group are essentially based on retrospective data and low-quality evidence; thus, follow-up recommendations are graded as weak in the current guidelines. Surveillance protocols for NMIBC tumours are based on regular cystoscopic examination and the use of urinary cytology, the current standard urine test used in clinical practice due to its excellent specificity and high sensitivity for HG disease detection [4,10]. Urine cytology is considered the gold-standard urine test for follow-up of high-risk NMIBC for the EAU and for both high- and intermediate-risk patients for the AUA in conjunction with cystoscopy. High-risk NMIBC also includes patients with LG tumours and additional risk factors according to the 2021 EAU NMIBC scoring model. However, in patients with high-risk disease but LG tumours, information regarding the usefulness of cytology during surveillance to detect recurrences is scarce, keeping in mind that urine cytology has a poor performance in detecting LG tumours [11,12]. Additionally, in patients diagnosed with LG BC or/and classified into the low-risk group, tumour recurrence tends to be of LG [13,14]. Therefore, urinary cytology may not add any value to cystoscopy during follow-up in this subgroup of patients. However, series show that there might be an overuse of cytology in the low-risk scenario or in patients diagnosed with LG tumours [7,8]. Matulay et al. found that 53% of the urologists from the SUO routinely use urinary cytology during surveillance of low-risk patients apart from cystoscopy. Bree et al. analysed data from 13,054 patients with low-grade Ta tumours and found urinary cytology or other urine markers were used during follow-up in up to 54.9% of patients diagnosed with LG Ta. To date, there are no publications assessing the complementary role of cytology and cystoscopy in a predominantly LG NMIBC population. Therefore, we assessed the role of bladder-washing cytology as a complementary method to cystoscopy for LG TaT1 BC follow-up, considering that in our institution between 2010 and 2020, we routinely performed urine cytology for NMIBC surveillance regardless of risk group.

We found that urine cytology only modified clinical management in three (0.89%) patients in our cohort of primary LG NMIBC. Similar conclusions were recently suggested by Feiertag et al. in a cohort of patients predominantly with intermediate- or high-risk BC with HG tumours. They found that in only 0.4% cases of their study population, the cytology result caused an escalation in clinical management, which suggested that this change in clinical management probably would not have any impact on BC progression or mortality [15]. Van der Aa et al. proposed that the use of urinary cytology as an adjunct to cystoscopy could increase cystoscopy accuracy and recurrence diagnosis by knowing the urine test outcome at the time of the cystoscopy due to the diagnostic review bias [16]. Nevertheless, its uncertain if these early detected recurrences have any clinical impact on a patient’s prognosis or could be amenable for expectant management, especially in LG scenarios. However, we could not assess this question as we perform cytology during cystoscopy.

In our study, all patients in which cytology changed the management had a previous suspicious cystoscopy. This supports the fact that performing bladder-washing urine cytology could be useful as a complementary method to cystoscopy when a suspicious area or lesion is seen, regardless of the risk group. In the case that the cytology is positive for HG UC, bladder biopsies, at least of the suspected area, are advised [17]. In fact, we diagnosed all three patients in which a positive cytology changed management after a suspicious cystoscopy with HG lesions after bladder biopsies, which supports this statement. On the other hand, we found no patients initially diagnosed with LG NMIBC with normal follow-up cystoscopies but positive cytology that led to recurrence diagnosis, which sustain guideline recommendations to avoid unnecessary urine cytology especially in the low-risk group. Furthermore, it is still uncertain how to proceed with positive cytologic findings despite negative cystoscopy and how this affects patients’ follow-up. It is suggested to repeat cystoscopic evaluation plus random bladder biopsies and, in the case of negative biopsies and persistent positive cytology, to perform upper urinary tract imaging [18].

Regarding the impact of urinary cytology as a prognostic factor of progression to HG disease in patients with initial LG NMIBC, it is not entirely known. In fact, prognostic models do not usually include cytology results, and most previous studies focused on the relation between positive cytology at diagnosis and subsequent risk for recurrence [19]. Concerning progression, Bell et al. state that a positive urinary cytology has no prognostic role in BC in terms of progression [20]. If we focus in LG NMIBC, Jackson et al. demonstrated, in a small cohort, that a positive cytology at diagnosis was associated with either recurrence or progression to HG urothelial carcinoma during follow-up. Similarly, in our study we found that a positive cytology at first recurrence is associated with a higher risk of progression to HG disease and with lower upgrade-free survival, suggesting that urinary cytology could have prognosis implications when detecting BC recurrence. Although further studies are needed, all these findings indicate that urine cytology has a limited role during follow-up of initial LG NMIBC; however, if recurrence is suspected, urine cytology might be useful for risk assessment during further follow-up.

We acknowledge some limitations in the present study. First, cytology was performed when the cystoscopy was done, so we could not evaluate the role of knowing the cytology result before the cystoscopy. Second, interobserver variability especially influences cystoscopy and cytology interpretation. Another limitation is that the current study did not evaluate the cost-effectiveness of using urine cytology for patients with LG NMIBC. Finally, we did not evaluate the role of urinary cytology performed after first BC recurrence.

## 5. Conclusions

The role of bladder-washing urine cytology as an adjunctive to cystoscopy to detect first recurrences during follow-up of primary LG TaT1 NMIBC might be limited to patients with non-conclusive suspicious lesions in the cystoscopy. In patients with initial LG TaT1 diagnosed with a first BC recurrence, a positive cytology result could alert to the risk of progression to HG urothelial carcinoma during further follow-up.

## Figures and Tables

**Table 1:** Characteristics of patients with LG NMIBC.

Variables	*n* = 337
Age at diagnosis (years), median (IQR)	68 (59–78)
Sex, *n* (%)	
Male	268 (79.5)
Female	69 (20.5)
Initial pT stage, *n* (%)	
Ta	299 (88.7)
T1	38 (11.3)
EAU NMIBC prognostic factor risk group, *n* (%)	
Low-Risk	262 (77.7)
Intermediate-Risk	57 (16.9)
High-Risk	18 (5.3)
Follow-up (years), median (IQR)	5.0 (3.6–6.7)
Disease Recurrence, *n* (%)	166 (49.3)

LG = Low-grade; NMIBC = Non-muscle-invasive bladder cancer; IQR = Interquartile range; EAU = European Association of Urology.

**Table 2:** Characteristics of patients with recurrence after initial LG NMIBC.

Variables	*n* = 166
Age at recurrence (years), median (IQR)	68 (59–78)
Sex, *n* (%)	
Male	134 (80.7)
Female	32 (19.3)
Initial pT stage, *n* (%)	
Ta	147 (88.6)
T1	19 (11.4)
EAU NMIBC prognostic factor risk group, *n* (%)	
Low-Risk	126 (75.9)
Intermediate-Risk	31 (18.7)
High-Risk	9 (5.4)
Time to Recurrence (months), median (IQR)	12 (6–23)
Cystoscopy findings, *n* (%)	
Negative	0 (0.0)
Suspicious lesion	12 (7.2)
Positive	154 (92.8)
Cytology results, *n* (%)	
Negative for UC	104 (62.7)
Atypia	9 (5.4)
Suspicious for UC	20 (12.1)
Positive for UC	13 (7.8)
Insufficient for diagnosis	20 (12.0)
Cytology results grouped, *n* (%)	
Negative	113 (68.1)
Positive	33 (19.9)
Insufficient for diagnosis	20 (12.0)

LG = Low-grade; NMIBC = Non-muscle-invasive bladder cancer; IQR = Interquartile range; EAU = European Association of Urology; UC = Urothelial carcinoma.

**Table 3:** Patients for whom cytology changed management (*n* = 3, 0.89%). yo = years old; HGUC = High-grade urothelial carcinoma; CIS = Carcinoma in situ.

Patient Characteristics	Cystoscopy at Recurrence	Initial Management	Cytology	Management Change	Recurrence Pathology
Male 76 yo Debut LGT1 EAU High Risk	Suspicious area	Early cystoscopic control	Positive for HGUC	Biopsy of the suspected area + mapping biopsies	CIS
Male 69 yo Debut LGTa EAU Low Risk	Suspicious lesion	Early cystoscopic control	Positive for HGUC	Biopsy of the suspected area + mapping biopsies	CIS
Male 65 yo Debut LGTa EAU Low Risk	Suspicious papillary lesion	Outpatient appointment to check cytology result	Positive for HGUC	TURBT + bladder biopsies	HGTx + CIS

## Data Availability

The data supporting the findings of this study are available upon reasonable request to the corresponding author.

## Supplementary Materials

The following supporting information can be downloaded at https://www.mdpi.com/article/10.3390/cancers16213708/s1, Table S1: Recurrence patterns; Table S2: Characteristics of patients with recurrence after initial LG NMIBC stratified by risk-group; Table S3: Abbreviation table; Figure S1: Examples of cystoscopic images categorized as suspicious according to the treating physician notes. In these cases, a positive bladder-washing cytology changed the management and diagnosed a recurrence.
