# Supplementary material for: The Role of Bladder-Washing Cytology as an Adjunctive Method to Cystoscopy During Follow-Up for Low-Grade TaT1 Non-Muscle-Invasive Bladder Cancer
Source: Cancers (Basel). 2024 Nov 1;16(21):3708. doi: 10.3390/cancers16213708 (PMC11545547; doi:10.3390/cancers16213708)
Supplement: Supplementary file 1 [file cancers-16-03708-s001.zip › cancers-3241029-supplementary.pdf]

## Supplementary Data

**Table S1.** Recurrence patterns.

| Variables | *N* = 166 |
|---|---|
| 1st Recurrence pT stage, n (%) | |
| Tx | 10 (6.0) |
| Tis | 6 (3.6) |
| Ta | 134 (80.7) |
| T1 | 15 (9.0) |
| T2 | 1 (0.6) |
| 1st Recurrence tumour grade, n (%) | |
| Low-grade | 148 (89.2) |
| High-grade | 18 (10.8) |
| Number of recurrences during follow-up, n (%) | |
| 1 | 75 (45.2) |
| 2 | 35 (21.1) |
| 3 | 25 (15.1) |
| 4 or more | 31 (18.7) |
| Upgrading or/and upstaging during follow-up, n (%) | 41 (24.7) |
| Time to Upgrading/Upstaging, months (IQR) | 18 (7.5-47.0) |
| Progression to HGUC, n (%) | 36 (21.7) |
| Time to HGUC, months (IQR) | 20.5 (8-49.75) |
| Upstaging, n (%) | 21 (12.7) |
| Time to Upstaging, months (IQR) | 15 (8-32.5) |
| Progression to MIBC, n (%) | 7 (4.2) |
| Time to MIBC, months (IQR) | 37 (15-44) |
| Radical cystectomy, n (%) | 8 (4.8) |

IQR = Interquartile range; HGUC = High grade urothelial carcinoma; MIBC = Muscle invasive bladder cancer

**Table S2.** Characteristics of patients with recurrence after initial LG NMIBC stratified by risk-group.

| Variables | *Low-Risk* | *Intermediate-Risk* | *High-Risk* | *p* |
|---|---|---|---|---|
| Patients, n (%) | 126 (75.9) | 31 (18.7) | 9 (5.4) | |
| Age at recurrence (years), median (IQR) | 67 (60- 74.3) | 79 (75-83) | 79 (76.5-82.0) | <0.001 |
| Sex, n (%) | | | | 0.738 |
| Male | 102 (81.0) | 24 (77.4) | 8 (88.9) | |
| Female | 24 (19.0) | 7 (22.6) | 1 (11.1) | |
| Initial pT stage, n (%) | | | | <0.001 |
| Ta | 118 (93.7) | 25 (80.6) | 4 (44.4) | |
| T1 | 8 (6.3) | 6 (19.4) | 5 (55.6) | |
| Time to Recurrence (months), median (IQR) | 13.5 (7.0-28.3) | 10 (6.0-19.0) | 7 (4.0-16-0) | 0.074 |
| Cystoscopy findings, n (%) | | | | 0.122 |

| | | | | |
|---|---|---|---|---|
| Negative | 0 (0.0) | 0 (0.0) | 0 (0.0) | |
| Suspicious lesion | 10 (7.9) | 1 (3.2) | 1 (11.1) | |
| Positive | 116 (92.1) | 30 (96.8) | 8 (88.9) | |
| Cytology results, n (%) | | | | 0.373 |
| Negative for UC | 78 (61.9) | 21 (67.7) | 5 (55.6) | |
| Atypia | 8 (6.3) | 1 (3.2) | 0 (0.0) | |
| Suspicious for UC | 14 (11.1) | 5 (16.1) | 1 (11.1) | |
| Positive for UC | 9 (7.1) | 1 (3.2) | 3 (33.3) | |
| Insufficient for diagnosis | 17 (13.5) | 3 (9.7) | 0 (0.0) | |
| Cytology results grouped, n (%) | | | | 0.443 |
| Negative | 86 (68.3) | 22 (71.0) | 5 (55.6) | |
| Positive | 23 (18.2) | 6 (19.3) | 4 (44.4) | |
| Insufficient for diagnosis | 17 (13.5) | 3 (9.7) | 0 (0.0) | |
| 1st Recurrence pT stage | | | | 0.030 |
| Tx | 7 (5.6) | 2 (6.5) | 1 (11.1) | |
| Tis | 5 (4.0) | 0 (0.0) | 1 (11.1) | |
| Ta | 106 (84.1) | 24 (77.4) | 4 (44.4) | |
| T1 | 7 (5.6) | 5 (16.1) | 3 (33.3) | |
| T2 | 1 (0.8) | 0 (0.0) | 0 (0.0) | |
| 1st Recurrence tumour grade | | | | 0.015 |
| LG | 116 (91.3) | 28 (90.3) | 5 (55.6) | |
| HG | 11 (8.7) | 3 (9.7) | 4 (44.4) | |
| Number of recurrences during follow-up | | | | 0.339 |
| 1 | 59 (46.8) | 13 (41.9) | 3 (33.3) | |
| 2 | 26 (20.6) | 8 (25.8) | 1 (11.1) | |
| 3 | 17 (13.5) | 7 (22.6) | 1 (11.1) | |
| 4 or more | 24 (19.1) | 3 (9.7) | 4 (44.4) | |
| Progression to HGUC, n (%) | 22 (17.5) | 9 (29.0) | 5 (55.6) | 0.018 |
| Time to HGUC, months (IQR) | 20.5 (10.25-50.25) | 26 (7.5-69.5) | 17 (6-20) | 0.367 |
| Upstaging, n (%) | 10 (7.9) | 7 (22.6) | 4 (44.4) | 0.002 |
| Time to Upstaging, months (IQR) | 12.5 (8.75-37.75) | 22 (8-26) | 17.5 (4.75-40.0) | 0.904 |
| Progression to MIBC, n (%) | 5 (4.0) | 0 (0.0) | 2 (22.2) | 0.036 |
| Time to MIBC, months (IQR) | 16 (13.5-44.0) | N/A | 41.5 (SD ± 3.5) | 0.245 |

LG = Low grade; NMIBC = Non-muscle invasive bladder cancer; IQR = Interquartile range; UC = Urothelial carcinoma; HG = High grade; HGUC = High grade urothelial carcinoma; MIBC = Muscle invasive bladder cancer

**Table S3.** Abbreviation table.

| Abbreviation | Abbreviated term |
|---|---|
| NMIBC | Non-muscle-invasive bladder cancer |
| LG | Low-grade |
| BC | Bladder cancer |
| HG | High-grade |
| EAU | European Association of Urology |

| | |
|---|---|
| AUA | American Urological Association |
| CIS | Carcinoma-in-situ |
| UC | Urothelial carcinoma |
| SUO | Society of Urologic Oncology |
| IRB | Institutional review board |
| TURBT | Transurethral resection of bladder tumour |
| UTUC | Upper tract urothelial carcinoma |
| BCG | Bacillus Calmette-Guérin |
| IQR | Interquartile ranges |
| HR | Hazard ratio |
| CI | Confidence interval |
| yo | years old |

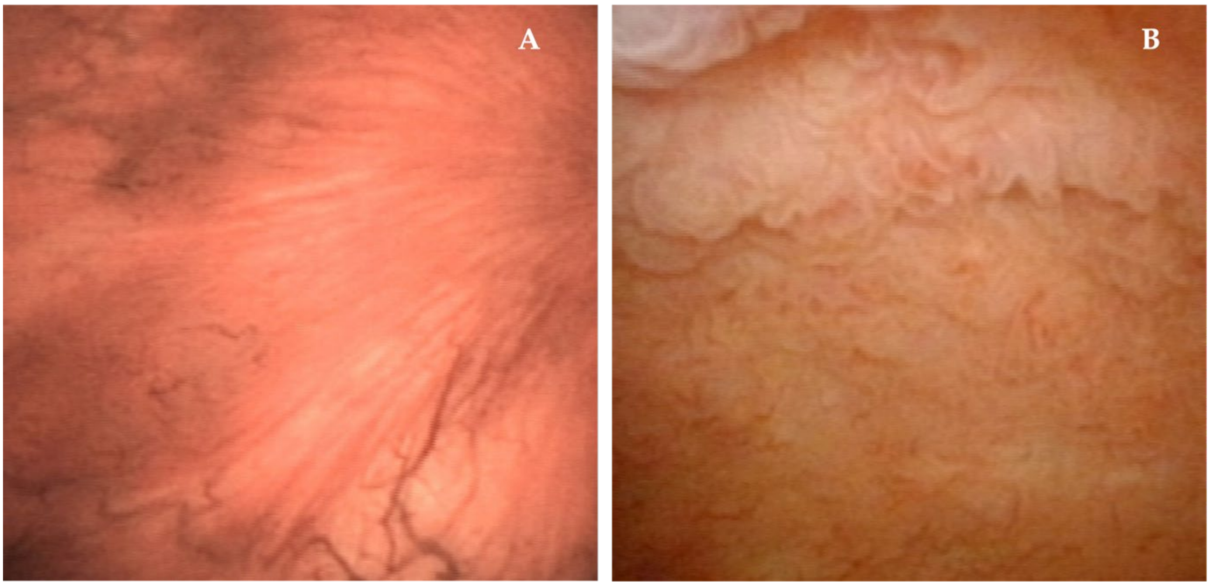


**Figure S1.** Examples of cystoscopic images categorized as suspicious according to the treating physician notes. In these cases, a positive bladder washing cytology changed the management and diagnosed a recurrence.
